# Managing Large Perforated Internal Root Resorption With Partial Pulpectomy: A Case Report

**DOI:** 10.1002/ccr3.70099

**Published:** 2025-01-09

**Authors:** Saeed Asgary

**Affiliations:** ^1^ Iranian Centre for Endodontic Research, Research Institute of Dental Sciences Shahid Beheshti University of Medical Sciences Tehran Iran

**Keywords:** calcium‐enriched mixture cement, internal root resorption, pulpitis, pulpotomy, vital pulp therapy

## Abstract

Internal root resorption (IRR) is a complex and often asymptomatic dental condition that can severely compromise tooth vitality and function. This case report presents the successful management of a perforated large IRR lesion in a 49‐year‐old female using an ultraconservative approach involving partial pulpectomy (PP) using calcium‐enriched mixture (CEM) cement. The patient, initially presenting with an asymptomatic resorptive lesion in her left first premolar, underwent ultraconservative PP following diagnosis via conventional radiography and cone beam computed tomography. Despite encountering excessive bleeding during treatment, the tampon technique using CEM cement allowed reasonable hemostasis and effective sealing of the pulp stump, facilitating the preservation of tooth structure. Follow‐up at 2 weeks and 2 years showed positive outcomes, including cessation of the resorptive process and normal periodontal health. This case demonstrates the efficacy of vital pulp therapy, specifically partial pulpectomy, in managing advanced IRR and underscores the potential for tooth preservation and long‐term stability through an ultraconservative approach.

**Summary**

- This case report highlights the successful management of a large perforated internal root resorption using a straightforward and minimally invasive partial pulpectomy with a tampon approach, employing an endodontic biomaterial.
- The treatment demonstrated favorable clinical and radiographic outcomes over a follow‐up period of 2 years.

## 1 Introduction

Internal root resorption (IRR) is a rare but significant pathological process characterized by progressive loss of dental hard tissues originating from within the root canal [1, 2]. This resorption is mediated by odontoclastic activity, where clastic cells degrade dentin along the inner root canal walls. IRR often remains asymptomatic and progresses slowly, making early detection challenging. It is typically diagnosed incidentally through routine radiographic imaging. Radiographs of IRR cases commonly reveal oval or round radiolucent areas within the pulp chamber or root canal space, indicating the characteristic enlargement of the affected area [3].

The exact etiology of IRR remains poorly understood, though it is considered multifactorial. Known contributing factors include dental trauma, recurrent or incipient carious lesions, periodontal infections, and thermal injury during procedures like crown preparations. Additionally, orthodontic movement, cracks, insufficient remaining dentin after preparation, marginal leakage from restorations, and idiopathic changes within otherwise healthy pulps have all been implicated in IRR development. Some researchers also suggest anachoresis, the attraction of microorganisms to inflamed tissue, as a contributing factor [4]. While IRR remains rare, its diagnosis and treatment pose significant challenges, especially given the limitations of two‐dimensional radiographs in detecting early lesions.

Histologically, IRR involves an inflammatory reaction within the pulp, disrupting the odontoblastic layer and predentin, facilitating the adhesion of clastic cells capable of resorbing dentin. Pulp inflammation may arise from infection or trauma, with inflammatory cells transported by the pulp's vascular supply. Odontoclastic cells, similar to osteoclasts, are responsible for resorption, although they are smaller and form less extensive lacunae. Two types of IRR are commonly described: Inflammatory IRR, characterized by dentin loss with a granulation tissue response, and replacement IRR, where resorption is accompanied by the deposition of hard tissue resembling bone or cementum [3].

The management of IRR primarily focuses on halting the resorptive process by eliminating the pulp's vascular supply, which nourishes the clastic cells. Nonsurgical root canal therapy (RCT) is the most common treatment approach, with early diagnosis being key to success [5]. Advancements in imaging, particularly with cone beam computed tomography (CBCT), now allow for more accurate assessment, enabling better visualization of the lesion's extent and location [6, 7].

Recent developments in endodontic biomaterials, such as mineral trioxide aggregate (MTA) and calcium‐enriched mixture (CEM) cement, have further improved prognosis in simple IRR cases [8, 9]. CEM cement is a biocompatible, tooth‐colored material known for its antibacterial, antifungal, and sealing properties. It also promotes osteogenesis, dentinogenesis, and cementogenesis, offering enhanced biological sealing and protection against microbial invasion [10].

This case report presents the ultraconservative management of a perforating IRR lesion in tooth #34 using partial pulpectomy (PP) and CEM cement. This approach effectively halts the resorptive process, preserving tooth structure and function, while offering a long‐term solution for managing advanced IRR.

## 2 Case History/Examination

**FIGURE 1:**
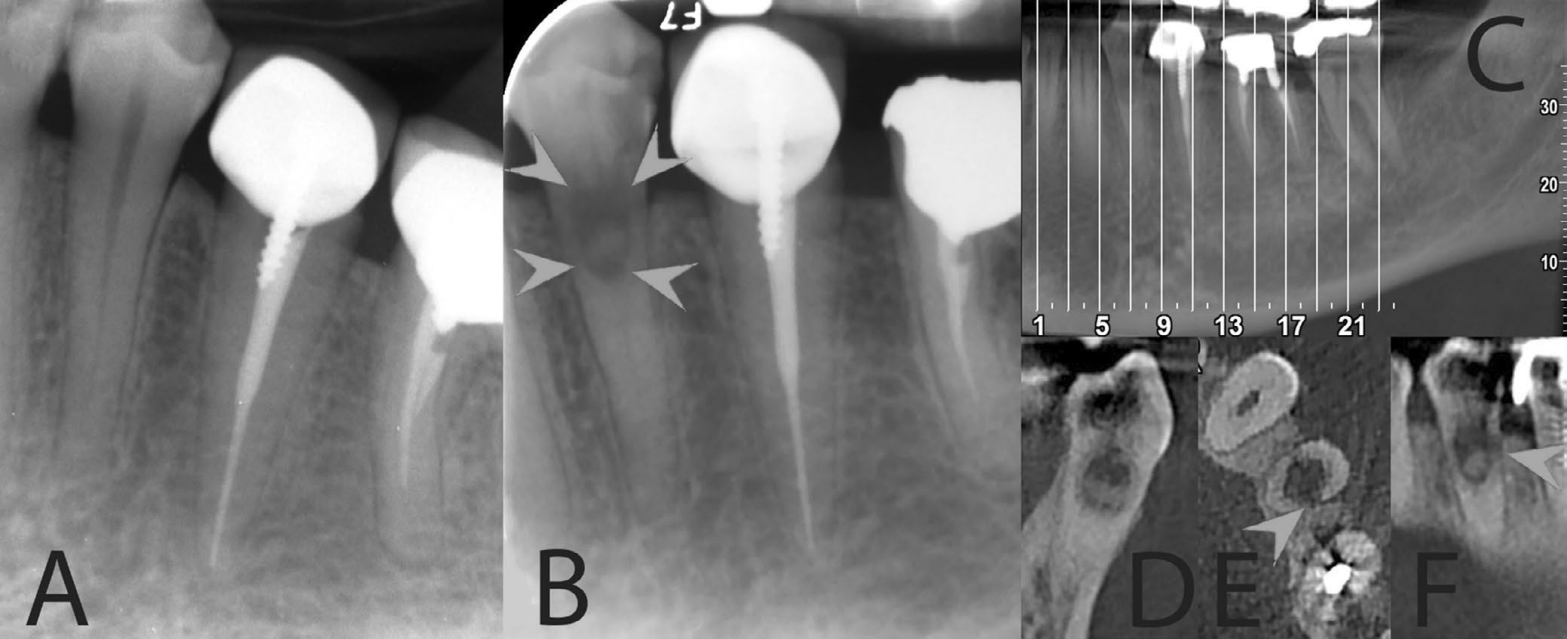
Radiographic and CBCT Images of Tooth #34. (A) Initial radiograph of the left first premolar taken 5 years prior, showing no signs of resorption or pathology. (B) Diagnostic periapical radiograph displaying a deep distal carious lesion and a significant resorptive defect in the coronal portion of the root of tooth #34 (white arrow heads). (C–F) CBCT images (in coronal, axial, and sagittal plans) of tooth #34, showing a large resorptive lesion extending into the midroot, with a distal perforation (white arrow heads) covered by surrounding bone. The lesion's extent is clearly demonstrated, aiding in accurate diagnosis and treatment planning.

A 49‐year‐old female patient was referred to our endodontic clinic following a routine dental check‐up that revealed an asymptomatic lesion on her left first premolar. Radiographs taken 5 years ago during a previous visit showed no abnormalities or signs of resorption (Figure 1A). At the time of referral, the patient was asymptomatic, reporting no pain, sensitivity, or discomfort in the affected tooth, except for occasional food impaction during mastication.

Upon clinical examination, the only significant finding was a carious lesion on the distal aspect of tooth #34. Sensibility testing on all regional teeth showed normal or positive responses, indicating pulp vitality. Notably, there was no history of orthodontic treatment or trauma to the tooth.

A diagnostic periapical radiograph revealed a deep distal carious lesion along with a substantial resorptive defect involving the coronal portion of the root of tooth #34 (Figure 1B). These findings prompted further investigation. A CBCT scan confirmed the presence of a large resorptive lesion extending into the mid‐root area, with a perforation on the distal aspect of the root (Figure 1C–F).

The clinical and radiographic findings led to the diagnosis of a perforated IRR with asymptomatic pulpitis in tooth #34. This indicated extensive resorption of the coronal dentin and pulp, with the involvement of the mid‐root region. The diagnosis of perforated IRR was established based on clinical and radiographic findings, differentiating it from external root resorption.

Treatment options were discussed, including surgical intervention, RCT, or tooth extraction. However, the patient opted for a conservative approach involving vital pulp therapy (VPT) with biocompatible materials. After thorough discussion, written informed consent was obtained from patient, and the treatment plan was finalized.

## 3 Methods

The procedure commenced with local anesthesia using 2% lidocaine with 1:80,000 epinephrine (DarouPakhsh, Tehran, Iran). The carious tissue was carefully removed, and an access cavity was prepared to expose the pulp and resorptive tissue. Upon exposure, the resorptive tissue was thoroughly debrided, but excessive hemorrhage was encountered.

Despite the application of normal saline for 2 min and 5.25% NaOCl for another 2 min, the bleeding persisted. To manage the bleeding and facilitate uninterrupted treatment, CEM cement (BioniqueDent, Tehran, Iran) was used as the pulp‐protecting and reparative biomaterial in a tampon approach [11]. The CEM cement was prepared according to manufacturer's instructions and inserted to fill/seal the remaining pulp stump and the prepared cavity (Figure 1E).

## 4 Results

**FIGURE 2:**
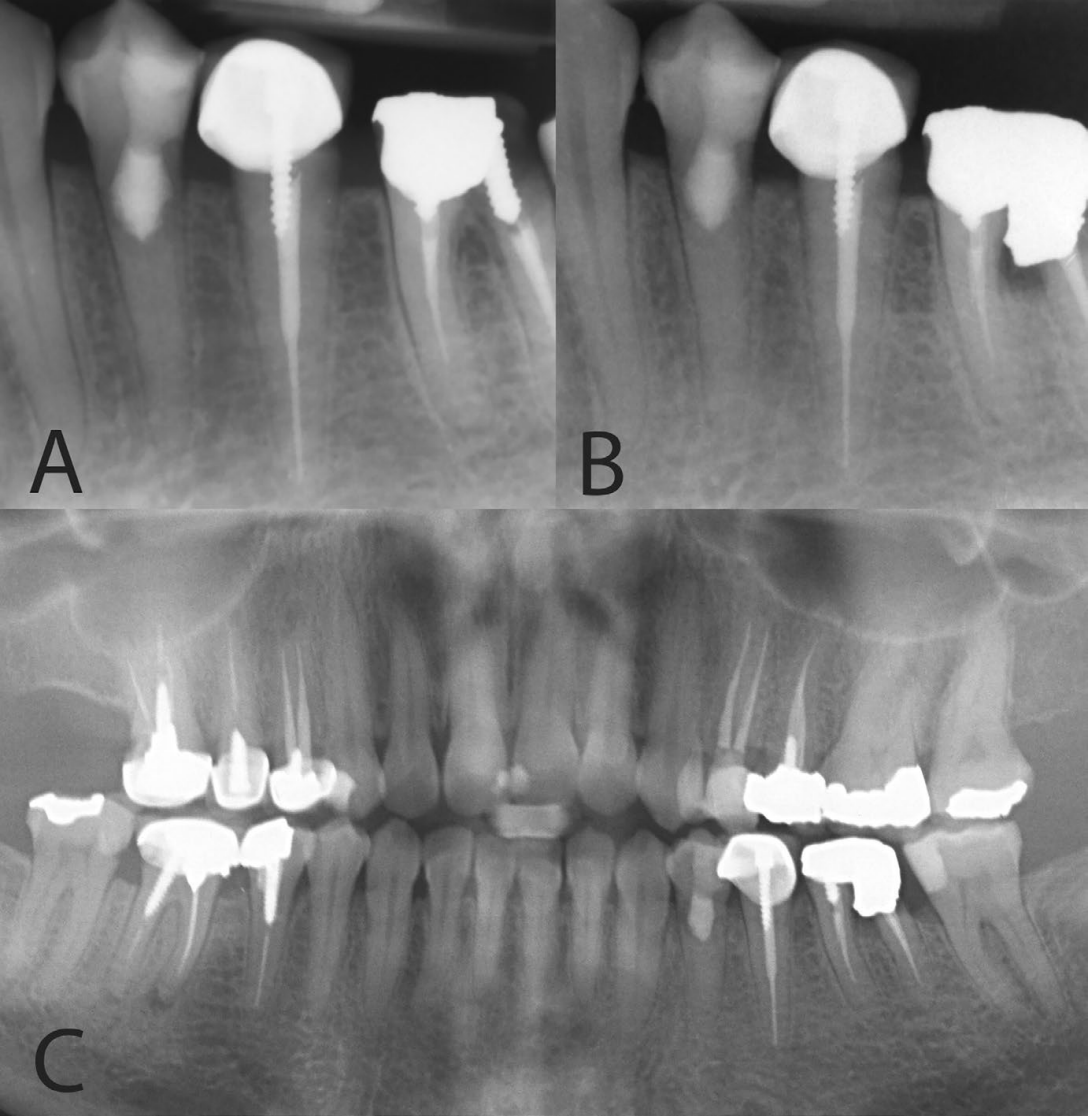
Treatment and follow‐up images of tooth #34. (A) Immediate postoperative periapical radiograph showing successful pulpotomy and proper sealing of the resorptive defect with CEM cement. (B) Two‐year post‐treatment radiograph revealing arrested resorption with a healthy periapical area of tooth #34. The periapical radiograph confirms the effectiveness of the treatment in halting further resorption. (C) OPG at the 2‐year recall, showing no signs of recurrence or additional resorption in the affected tooth. The radiograph highlights the stability of the treated tooth and surrounding structures.

An immediate postoperative periapical radiograph was obtained to confirm the success of the PP and the proper sealing of the resorptive defect (Figure 2A). The coronal cavity was then restored with composite resin to re‐establish the tooth's function and esthetic appearance.

The patient was reviewed periodically over a 2‐year follow‐up. One week post‐treatment, the patient remained asymptomatic, and the tooth was functional. At the 2‐year recall, the patient reported no discomfort or discoloration. Radiographic examination at the recall visit showed arrested resorption with a healthy periapical area and normal PDL, confirming the long‐term success of the treatment (Figure 2B,C).

## 5 Discussion

Root resorption, particularly IRR, is a rare yet significant condition that can severely compromise tooth vitality and structural integrity. In this case, a 49‐year‐old female presented with an asymptomatic lesion in the left first mandibular premolar, which was diagnosed as perforated IRR with asymptomatic pulpitis based on clinical and radiographic evaluation. IRR, characterized by the progressive loss of dentin and potential involvement of the pulp, presents a challenge for management, especially when extensive resorption with perforation has occurred.

The diagnosis of IRR in this patient was made following a comprehensive diagnostic workup, including conventional radiographs and advanced imaging with CBCT. The use of CBCT was crucial in assessing the extent of the resorptive lesion, which extended into the mid‐root with a distal perforation, aiding in the decision‐making process for treatment. This underscores the importance of advanced imaging in complex IRR cases, providing a more accurate assessment of lesion size, location, and the involvement of surrounding structures, which are vital in formulating an appropriate treatment plan [12].

Treatment options for complex IRR typically range from more invasive approaches, such as surgical intervention or RCT, to extraction, depending on the severity [13]. However, recent advances in regenerative endodontics and the use of biocompatible materials offer promising conservative alternatives. In this case, the patient opted for an ultraconservative approach involving PP with CEM cement. CEM cement is a biomaterial based on calcium‐silicate; it is highly bioactive and biocompatible with excellent mechanical properties for endodontic use [10]. CEM cement is manufactured using calcium oxide, phosphorus, and silicon. Upon hydration, it forms a colloidal gel that sets rapidly, is converted into hydroxyapatite, and consequently provides excellent tissue integration and mineralization. Its sealing properties avoid microleakage and bacterial penetration, essential for successful procedures [10]. According to the manufacturer, the biomaterial's capability to set in a wet environment suits its use for all VPT techniques, root‐end surgery, repair of root/furcal perforations, and management of internal/external root resorption.

CEM cement is a calcium‐silicate‐based material similar to MTA, inducing the formation of hydroxyapatite and thus supporting its biocompatibility [14]. It has a smoother consistency, greater flowability, thinner film thickness, and a shorter setting time [15]; hence, it improves handling and adaptability by streamlining clinical workflows. Its minimal positive dimensional changes when set reduce microleakage [15], an important attribute for the long‐term success of the restoration. Whereas MTA is still the gold standard, the profound advantages of CEM cement make it a very valuable alternative for both routine and complex endodontic procedures.

In this case, due to time constraints, the access cavity was filled during the same appointment. This is contrary to the recommendations of the manufacturer for a delay before the permanent restoration in order to allow the material to be set completely. However, an immediate restoration did not seem to compromise the clinical outcomes. This demonstrates the robustness and adaptability of the biomaterial in less‐than‐ideal clinical situations; however, adherence to guidelines is recommended for reproducibility.

This case demonstrates the potentials of CEM cement in ultraconservative endodontics, showing its efficacy for the achievement of hemostasis, sealing of resorptive defects, and maintenance of tooth vitality. Protocols should be tailored for the clinical scenario, recognizing their limitations. The results are in agreement with the literature that supports the use of CEM cement in the treatment of (perforated) internal/external root resorption [8, 16, 17, 18, 19]. Its regenerative potential, ease of handling, and durable sealing properties make it an important tool in contemporary endodontic practice, though further research is required to confirm its long‐term success and wider applicability.

Despite encountering excessive hemorrhage due to exposure of resorptive tissue, the tampon technique using CEM biomaterial effectively achieved hemostasis, allowing the procedure to proceed without delay [20, 21]. The biomaterial sealed the pulp stump and resorptive defect, facilitating immediate hemostasis through physical pressure and ensuring pulp preservation [9]. Postoperative radiographs confirmed a successful filling and sealing, and the patient remained asymptomatic. These findings align with previous studies that demonstrate the tampon technique with CEM cement provides reliable sealing in VPT, effectively managing resorptive defects and preventing further tooth damage. The tampon technique has been shown to yield favorable clinical and radiographic outcomes, including symptom resolution and arrest of the resorptive process, offering a promising alternative to conventional methods, particularly in cases complicated by hemorrhage [9].

The 2‐year follow‐up revealed that the resorption had arrested, with no signs of recurrence or further damage to the periapical tissues. Radiographic findings showed a healthy periapical area with no evidence of continued resorption, confirming the success of this conservative approach [22]. These results are consistent with similar cases in the literature, where vital pulp therapy has effectively halted internal resorption progression, preserved tooth vitality, and avoided more invasive treatments.

Although this case demonstrates successful outcomes, it is important to recognize that conservative treatments may not be suitable for all cases. The decision to pursue such approaches should be carefully evaluated, considering the extent of resorption, the patient's preferences, and the availability of biocompatible materials. Additionally, patient compliance and long‐term monitoring are crucial to ensuring continued effectiveness and stability. The lack of a control group and the limited sample size in this case highlight the need for further research, including larger case series and randomized controlled trials, to validate the efficacy and long‐term success of ultraconservative methods for IRR management.

## 6 Conclusions

This case illustrates that with appropriate diagnosis, advanced imaging, and biocompatible materials like CEM cement, conservative treatment options for IRR can be highly effective. Vital pulp therapy, particularly partial pulpectomy, offers a promising approach to managing IRR and preserving tooth vitality, especially in patients who are not candidates for more invasive treatments. Further long‐term studies are needed to validate the success rates of ultraconservative approaches and expand their application in clinical practice.

## Author Contributions

**Saeed Asgary:** conceptualization, data curation, investigation, methodology, resources, validation, visualization, writing – original draft, writing – review and editing.

## Consent

Written informed consents were obtained from the patients to publish this case series in accordance with the journal's patient consent policy.

## Conflicts of Interest

The author declares no conflicts of interest.

## Data Availability

The data used to support the findings of this study are included within the article.
